# Magnetic Resonance Imaging as a Complementary Diagnostic Tool for Aplastic Anemia

**DOI:** 10.1002/ajh.70236

**Published:** 2026-02-14

**Authors:** Jeanette Walter, Peter Isfort, Susanne Isfort, Jens Panse, Katharina Lindemann‐Docter, Tim H. Brümmendorf, Fabian Beier

**Affiliations:** ^1^ Department of Hematology, Oncology, Hemostaseology and Stem Cell Transplantation, Medical Faculty RWTH Aachen University Aachen Germany; ^2^ Center for Integrated Oncology – Aachen Bonn Cologne Duesseldorf (CIO‐ABCD) Aachen Germany; ^3^ Division of Hematological Malignancies, Department of Medical Oncology Dana Farber Cancer Institute Boston Massachusetts USA; ^4^ Center of Radiology, Neuroradiology and Interventional Radiology Diakovere Krankenhaus gGmbH Hannover Germany; ^5^ Department of Hematology, Hemostasis, Oncology and Stem Cell Transplantation, Comprehensive Cancer Center Lower Saxony Hannover Medical School Hannover Germany; ^6^ Department of Pathology University Hospital RWTH Aachen Aachen Germany

Acquired aplastic anemia (AA) is a rare, in most cases autoimmune‐mediated bone marrow failure syndrome characterized by pancytopenia and bone marrow (BM) hypocellularity below 25% [1]. Timely diagnosis is crucial to initiate immunosuppressive therapy and prevent life‐threatening complications. The diagnostic workup requires the exclusion of various differential diagnoses ranging from substrate deficiencies, viral infections, and malignant BM infiltration to myelofibrosis, hypoplastic myelodysplastic syndrome (MDS), and inherited BM failure syndromes [2, 3]. Histopathological investigation of BM biopsy is the gold standard to assess BM cellularity. Clinical interpretation of BM findings in AA can be challenging as BM may attenuate inhomogeneously, exhibiting a pattern of intermixed hypocellular and residual normocellular regions. In these cases, sampling bias can lead to conflicting results of low peripheral blood counts and normo‐ to hypercellular BM, which delays diagnosis and treatment. As previously reported, inhomogeneous BM attenuation in AA can be visualized as “patchy marrow pattern” in pelvic magnet resonance imaging (MRI), where residual hematopoietic islands appear as “patches” in an otherwise fatty BM [4, 5, 6, 7]. Although not being part of the standard diagnostic algorithm, the following case demonstrates how pelvic MRI served as a complementary diagnostic tool in a patient with strong suspicion of AA and discrepant BM findings.

We report the case of a 61‐year‐old woman in whom pancytopenia was incidentally discovered prior to elective polypectomy. The initial BM biopsy showed a mild hypocellularity (40%) affecting all hematopoietic lineages with pronounced megakaryo‐ and granulocytopenia, as well as dysplastic megakaryopoiesis not meeting the criteria for hypoplastic MDS or AA. Cytogenetics showed a normal female karyotype. Molecular analysis showed no variants associated with MDS. A maximum paroxysmal nocturnal hemoglobinuria (PNH) clone of 1.5% was identified. The patient was referred to our center for further examination for AA‐PNH overlap syndrome and exclusion of hypoplastic MDS.

Upon presentation, peripheral blood counts met the criteria for severe AA [1] (platelets 17 × 10^9^/L, neutrophils 0.43 × 10^9^/L, reticulocytes 47 × 10^9^/L, and hemoglobin 8 g/dL). In‐house BM biopsy showed a hypercellular BM with trilineage hematopoiesis (Figure 1A), prominent erythropoiesis, and erythroid dysplasia suggestive of MDS. To further investigate these discrepant BM findings, bilateral BM biopsy was taken, which revealed a hypo‐ to aplastic BM (cellularity < 20%) with absent granulo‐ and megakaryopoiesis, and residual focal erythropoiesis without dysplasia in both cases (Figure 1B). Given the contradictory BM biopsy results, pelvic MRI was performed.

**FIGURE 1:**
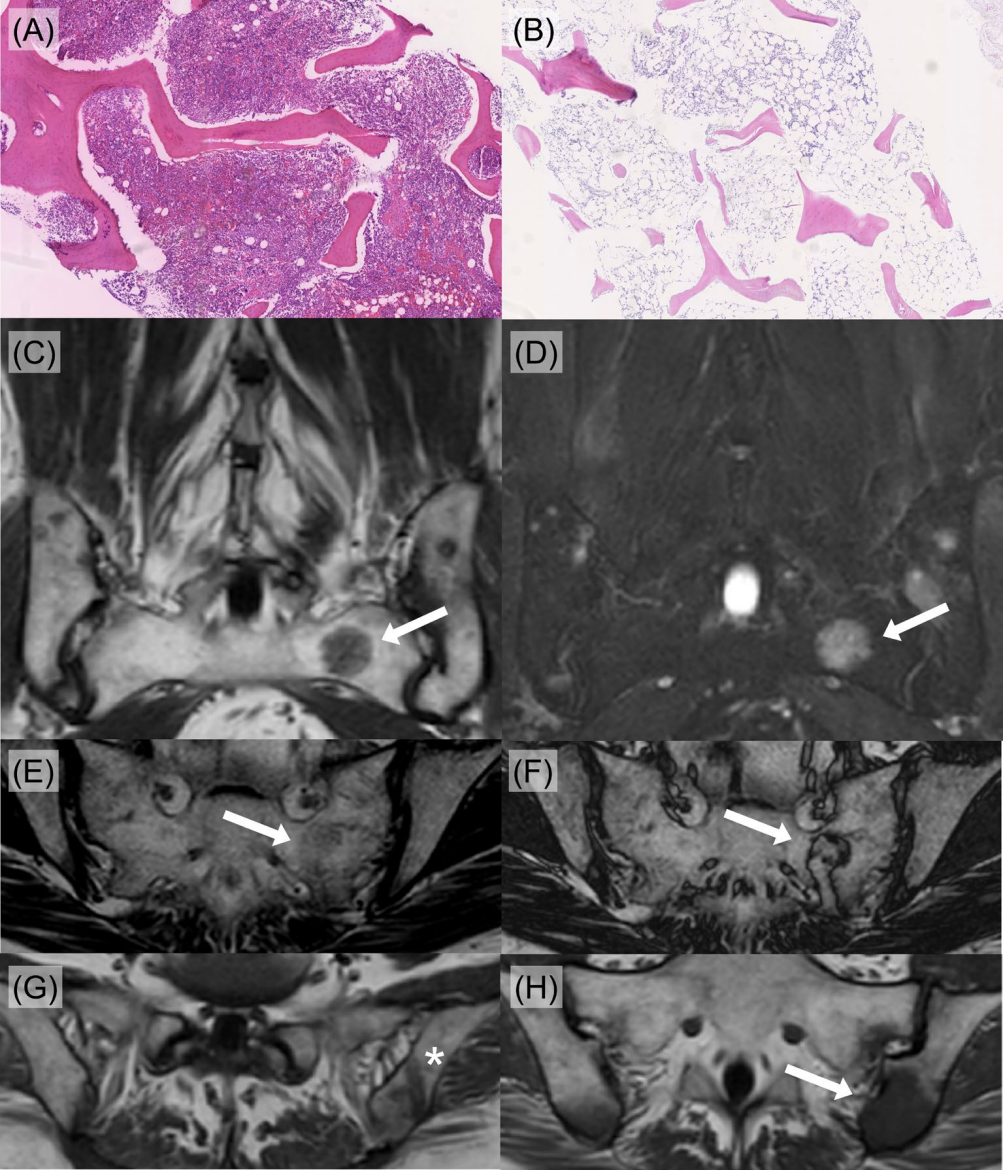
Histopathological findings and pelvic MRI scans. (A, B) Bone marrow (BM) biopsies: (A) The first in‐house BM biopsy showed markedly hypercellular marrow. (B) The second in‐house BM biopsy demonstrated hypocellularity (< 20%; hematoxylin–eosin staining, 5× magnification). (C, D) Coronal pelvic MRI: The characteristic “patchy marrow pattern” reflecting heterogeneous BM attenuation with residual hematopoietic islands (arrow). (C) T1‐weighted Turbo Spin Echo (TSE). (D) T2‐weighted TSE with spectral presaturation and inversion recovery (SPIR). (E–H) Axial pelvic MRI: (E, F) T1‐weighted Gradient Echo (GRE), in‐ and opposed‐phase. White arrows mark active BM. (G, H) T1‐weighted TSE: (G) Biopsy site and needle track (asterisk) of the residual hematopoietic island showing compensatory focal hyperplasia. (H) A residual BM island (white arrow) lies in close vicinity to the biopsy site shown in (G) (asterisk).

The MRI revealed a diffuse signal alteration in the BM, indicating an increased fat content alongside multiple “patches” of residual hematopoietic activity. These findings were consistent with compensatory focal hematopoiesis and the previously described “patchy‐marrow pattern.” Through T1‐weighted imaging without fat suppression, hematopoietic islands can be very well depicted as hypointense foci surrounded by regular hyperintense fatty bone marrow (Figure 1C). Consequently, in T2‐weighted imaging with fat suppression, lesions appear hyperintense with surrounding hypointense (suppressed) fatty bone marrow (Figure 1D). The site of the hypercellular BM biopsy could be traced and localized within a residual hematopoietic island, resolving the discrepancy within histologic findings (Figure 1E–H). Based on the integrated diagnostic assessment, the diagnosis of severe AA was confirmed. Immunosuppressive therapy with anti‐thymocyte globulin and cyclosporine was promptly initiated, leading to partial remission after 3 months and complete remission after 7 months.

This case demonstrates that pelvic MRI can provide crucial diagnostic information in cases of suspected AA with discordant BM findings. In selected patients, pelvic MRI can guide targeted BM biopsy [8]. Incorporating pelvic MRI into the standard diagnostic pathway may facilitate timely and accurate diagnosis of AA and prevent delays in initiating life‐saving therapy.

## Funding

This work was supported by the Mildred‐Scheel scholarship awarded to Jeanette Walter by the German Cancer Aid (“Deutsche Krebshilfe,” No. 5780246).

## Ethics Statement

The patient provided informed consent to the German aplastic anemia‐bone marrow failure registry (AA‐BMF) and consent for publication for this case report.

## Conflicts of Interest

The authors declare no conflicts of interest.

## Data Availability

Data sharing not applicable to this article as no datasets were generated or analyzed during the current study.
